# BTLA biology in cancer: from bench discoveries to clinical potentials

**DOI:** 10.1186/s40364-024-00556-2

**Published:** 2024-01-17

**Authors:** Anna Andrzejczak, Lidia Karabon

**Affiliations:** Laboratory of Genetics and Epigenetics of Human Diseases, Ludwik Hirszfeld Institute of Immunology and Experimental Therapy, Polish Academy of Sciences, Wroclaw, Poland

**Keywords:** Immune checkpoints, B and T lymphocyte attenuator (BTLA), Soluble BTLA (sBTLA), Cancer, Gene expression, Disease risk, Prognostic factor, Immune checkpoint blockade, Single nucleotide polymorphism (SNP)

## Abstract

Immune checkpoints play a critical role in maintaining the delicate balance of immune activation in order to prevent potential harm caused by excessive activation, autoimmunity, or tissue damage. B and T lymphocyte attenuator (BTLA) is one of crucial checkpoint, regulating stimulatory and inhibitory signals in immune responses. Its interaction with the herpes virus entry mediator (HVEM) plays an essential role in negatively regulating immune responses, thereby preserving immune homeostasis. In cancer, abnormal cells evade immune surveillance by exploiting checkpoints like BTLA. Upregulated BTLA expression is linked to impaired anti-tumor immunity and unfavorable disease outcomes. In preclinical studies, BTLA-targeted therapies have shown improved treatment outcomes and enhanced antitumor immunity. This review aims to provide an in-depth understanding of BTLA’s biology, its role in various cancers, and its potential as a prognostic factor. Additionally, it explores the latest research on BTLA blockade in cancer immunotherapy, offering hope for more effective cancer treatments.

## Introduction

The immune system acts as a natural defense against infections and cancer. It recognizes pathogens and abnormal cells, eliminating them in order to prevent disease progression. Immune checkpoints (ICs) maintain immune balance through co-stimulatory and co-inhibitory molecules on immune cell surfaces. An imbalance in these signals can lead to various diseases, including autoimmune disorders, infections, and cancer. Co-stimulatory ICs like CD28, CD40, and OX40 activate T-cells, while co-inhibitory ICs like CTLA-4 and PD-1 attenuate T-cell activation and proliferation. Understanding and targeting these ICs holds promise for developing effective treatments for a range of diseases [1].

The importance of ICs in cancer development and progression has been demonstrated by the remarkable success of antitumor treatments based on ICs blocking [2, 3]. Despite the impressive success of IC inhibitors in cancer treatment, not all patients respond to this therapy [4]. As a result, scientists continue to study the biology and functions of multiple ICs and their role in cancer development and progression, searching for new potential targets for immunotherapy. Among them, BTLA (B and T lymphocyte attenuator), an inhibitory IC, has recently gained attention as a promising therapeutic target for immunotherapy [5]. BTLA binding HVEM, acts as a negative modulator of the immune response by inhibiting T and B-cell activation, proliferation, and proinflammatory cytokine production [6]. Abnormal BTLA expression has been observed in various cancers, and has been linked to unfavorable disease outcomes [7, 8]. However, BTLA-HVEM signaling pathways are still poorly understood and require further investigation. In this review, our aim is to summarize the current state of knowledge concerning the biology of BTLA, its role in cancer immunosurveillance, and its potential as a prognostic factor in various cancers.

## BTLA biology

### BTLA structure

**Fig. 1:**
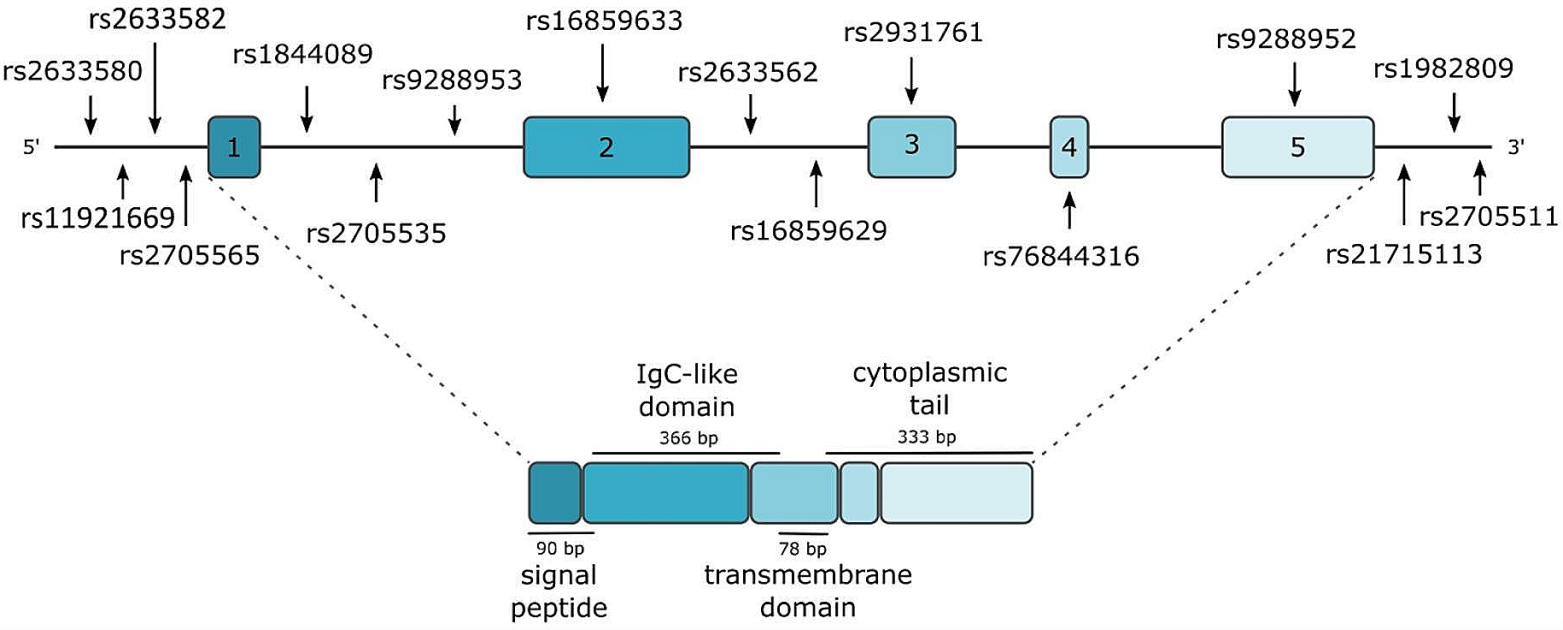
Structure of the *BTLA* gene and the BTLA protein along with the location of the studied *BTLA* gene variants. Blue boxes indicate exons and lines introns and untranslated regions of the gene

BTLA (also known as CD272) was discovered 20 years ago initially as a transcript, highly specific to Th1 cells [9]. The *BTLA* gene is located on chromosome 3 in q13.2 in reverse orientation and encodes a 289 amino acid long type 1 transmembrane glycoprotein (33 kD). The BTLA protein belongs to the CD28 immunoglobulin superfamily (IgSF) and shares structural and functional similarities with PD-1 and CTLA-4. The BTLA protein consists of a signal peptide, an IgC-like extracellular domain, a transmembrane domain, and a cytoplasmic domain [10] (Fig. 1). The BTLA protein is also produced in a soluble form (sBTLA) as a result of alternative RNA splicing. sBTLA lacks the transmembrane region, due to the lack of exon 3, however, the exact mechanism of sBTLA production has not been described yet [10].

### BTLA ligands

The herpes virus entry mediator (HVEM) is the first known BTLA ligand expressed in humans. Unlikely for CTLA-4 and PD-1 ligands, HVEM belongs to the tumor necrosis factor receptor (TNFR) superfamily [11]. However, BTLA is not a unique binding partner for HVEM. Alongside BTLA, CD160, LT-a, TNF-SF, and LIGHT can also bind to HVEM. CD160 competes with BTLA for the same binding site within the complementarity-determining region (CDR) 1 of HVEM, while LIGHT binds to the opposite side of HVEM within CRD2/CRD3 regions. Therefore, inhibitory and stimulatory ligands of HVEM bind at distinct sites [12, 13]. BTLA binding HVEM acts as a negative modulator of the immune response [14]. N-terminal cysteine-rich domain CRD1 of HVEM binds to a single IgC domain of BTLA presented on the cell surface in 1:1 stoichiometry [15]. The BTLA-HVEM complex is formed by interactions between 15 residues of BTLA and 12 residues of HVEM and mutations in those positions have a significant impact on BTLA-HVEM binding [13].

A few years ago, the UL144 viral protein was identified as the second BTLA ligand. The human cytomegalovirus opening reading frame UL144 is an ortholog of HVEM, sharing 36% amino acid sequence homology [16]. UL144 proteins activate similar inhibitory signaling pathways by inhibition of TCR-activation, and IFN/IL-2 signaling in B-cells and NK-cells [16]. Interestingly, despite the 5-fold lower affinity of UL144 to BTLA, UL144 inhibits T-cell proliferation to a greater extent than HVEM [12].

### BTLA signaling

**Fig. 2:**
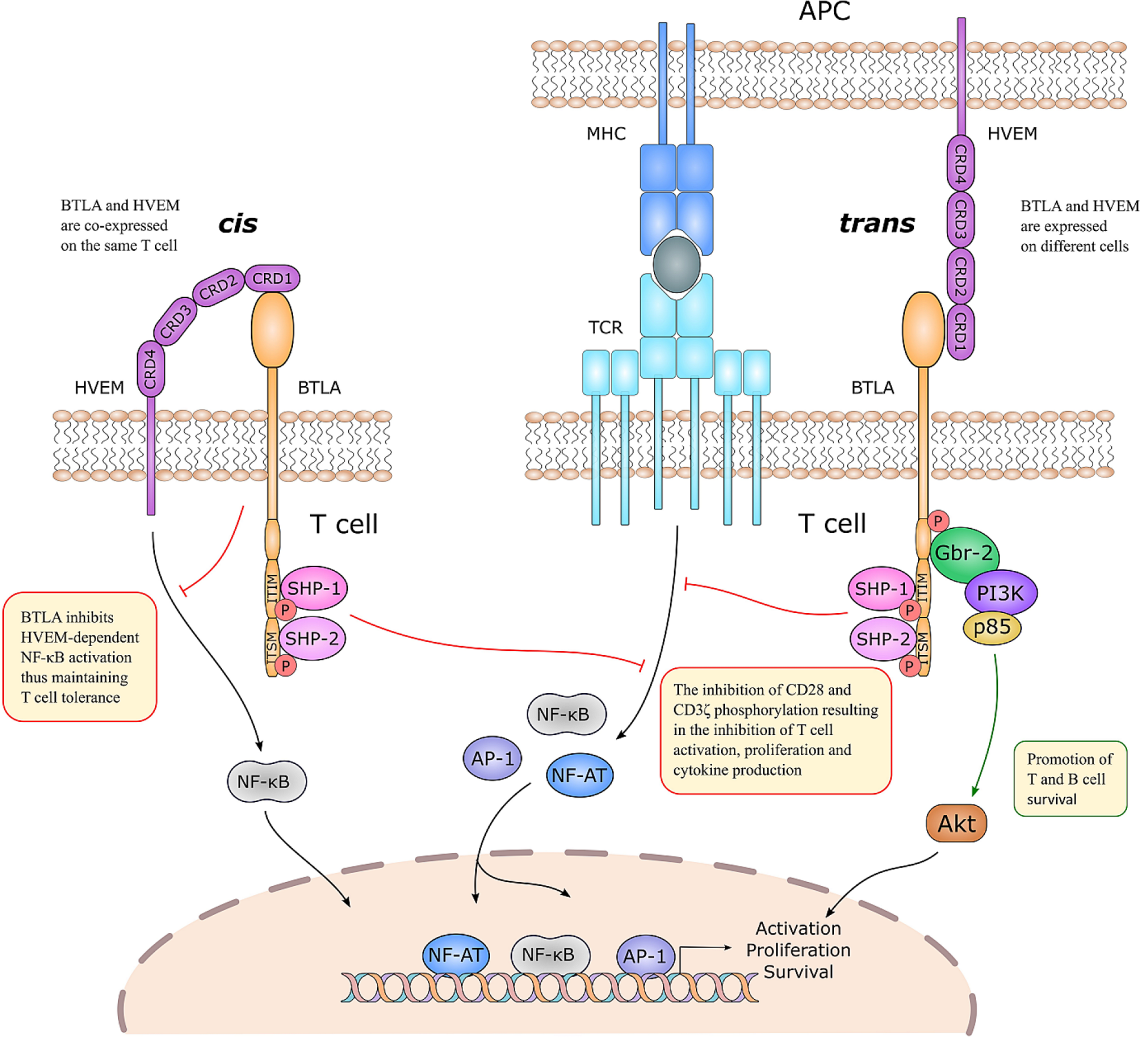
BTLA interacts with HVEM in a *cis* and *trans* manner. Left panel: *cis* interactions. BTLA and HVEM are expressed on the same cell. The BTLA-HVEM *cis* interaction prevents BTLA or HVEM from interacting with other co-signaling molecules in a *trans* manner and inhibits HVEM-dependent NF-κB activation in the T-cell promoting tolerance. Right panel: *trans* interactions. BTLA and HVEM are expressed on different cells. The BTLA-HVEM *trans* interaction provides bidirectional signaling in T-cells. BTLA engagement leads to the recruitment of SHP-1 and SHP-2, thereby downregulating the TCR signaling and providing inhibitory signals. Conversely, binding of the GRb-2 protein leads to the activation of the PI3K/Akt signaling promoting T-cell survival

The cytoplasmic region of BTLA comprises three highly conserved tyrosine-containing motifs; a growth-factor receptor-bound protein 2 (Grb2) binding site, an immunoreceptor tyrosine inhibitory motif (ITIM), and an immunoreceptor tyrosine-based switch motif (ITSM) [6, 17]. The presence of both inhibitory ITIM/ITSM motifs and stimulatory Grb-2 binding site makes BTLA a unique IC receptor with possible bi-directional signaling. After BTLA-HVEM crosslinking, tyrosine residues in both the ITIM and ITSM motifs are phosphorylated. This phosphorylation is necessary for the recruitment of tyrosine phosphatases SHP-1 and SHP-2, leading to downstream signaling activation. Either ITIM tyrosine (Y257) or ITSM tyrosine (Y282) are essential for the binding of both SHP-1 and SHP-2 by BTLA. Creation of the BTLA-SHP-1 complex leads to the inhibition of both CD28 and CD3ζ phosphorylation resulting in the inhibition of T-cell activation [18, 19]. On the other hand, the Grb-2 protein binding to the Grb-2 motif recruits the PI3K kinase subunit p85, leading to activation of the PI3K/Akt signaling pathway, which promotes cell activation and proliferation. This interaction suggests the pro-survival function of BTLA (Fig. 2) [17]. It is interesting that recent studies have demonstrated that BTLA on antigen presenting cells (APC) could also act as a costimulatory ligand for HVEM expressed on CD8 + T-cells by activation of the NF-κB signaling in T-cells and promoting proliferation of those cells [20].

HVEM can interact with BTLA not only in a *trans* but also in a *cis* manner (Fig. 2) when the immune system is resting. *Cis* signaling takes place when BTLA and HVEM are co-expressed on the same T-cell leading to the formation of BTLA-HVEM heterodimers. In this situation, BTLA binding represses HVEM co-stimulation, leading to the inhibition of HVEM-dependent trans-activating NF-κB signaling. This, in turn, decreases T-cell activation and cytokine production, thereby maintaining T-cell tolerance. Upon T-cell activation, HVEM levels diminish due to its temporary internalization and BTLA expression transiently increases, allowing BTLA and HVEM to interact with their ligands in a *trans* manner. On the contrary, BTLA signaling is not impaired when binding HVEM in a *cis* manner. This observation indicates the presence of a functional hierarchy where BTLA co-inhibition dominates over HVEM co-stimulation [20–22].

### BTLA expression

**Fig. 3:**
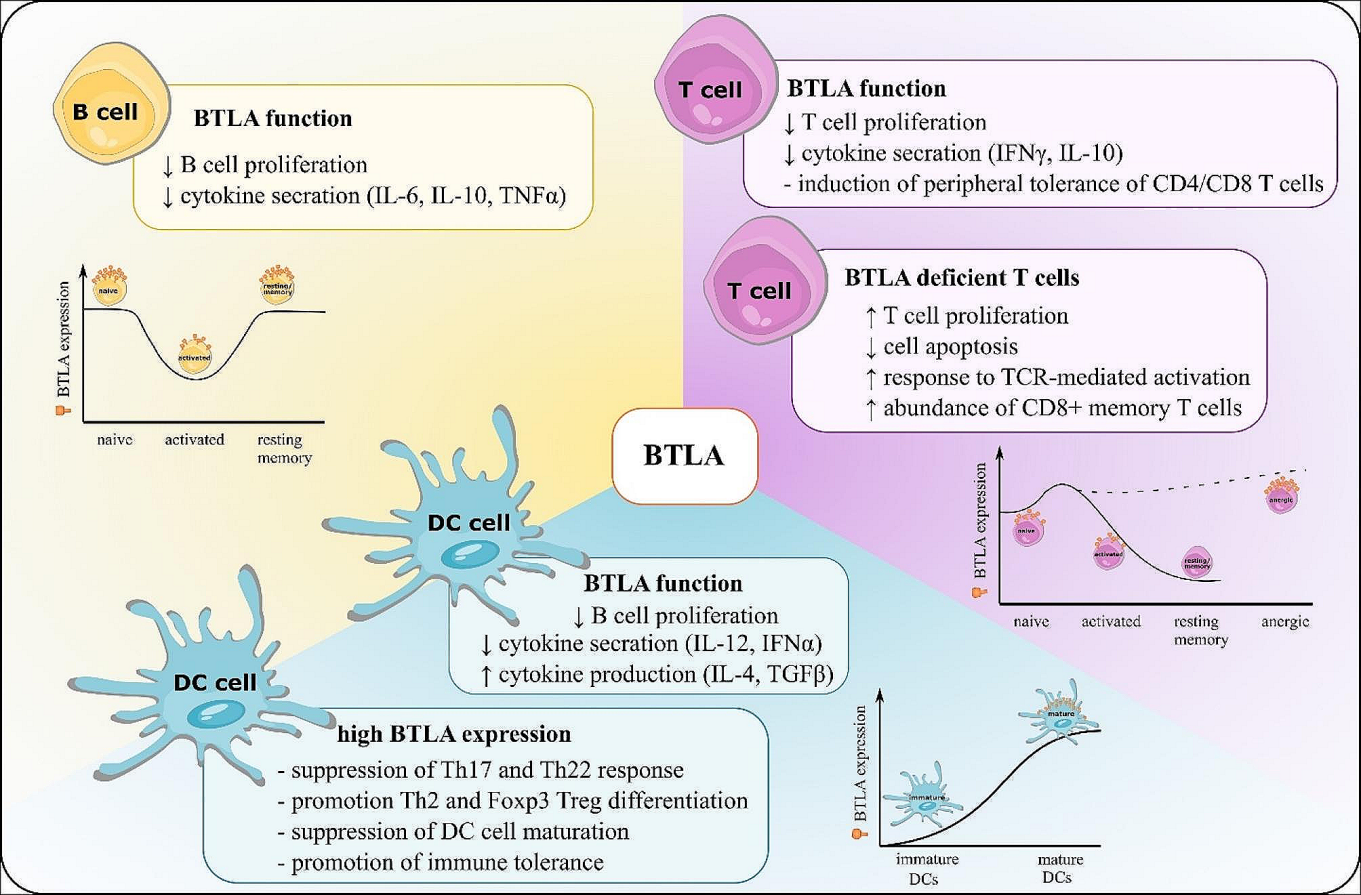
Summary of BTLA functions and expression dynamics in T cells, B cells, and DCs

In humans, the BTLA protein is presented almost exclusively on the surface of lymphoid and myeloid cells (Fig. 3), including CD4 + and CD8 + T-cells, NK-cells, B-cells, dendritic cells (DC), and macrophages. Tissue distribution shows high BTLA expression levels in lymph nodes, thymus and spleen [8]. Within freshly isolated peripheral blood mononuclear cells (PBMCs), BTLA + CD3 + constitute approximately 80% of T-cells and BTLA + CD19 + about 90% of B-cells [23–26].

### T-cells

BTLA expression can be detected from the early stages of T-cell differentiation. In the thymus, BTLA can be detected on T-cells during positive selection. BTLA is constitutively expressed on the majority of CD4 + and CD8 + T-cells and to a lesser extent on naïve T-cells, NK and NKT-cells, whereas regulatory T-cells express very low BTLA levels. Conversely, a high BTLA level is a characteristic feature of anergic T-cells and T follicular helper (Tfh) cells [27–30]. BTLA level is also dependent on the T-cell activation status, where BTLA expression transiently increases upon activation in naïve T-cells and decreases in activated T-cells [23–26]. Studies have shown that BTLA is not essential for proper T-cell development since BTLA-deficient mice exhibit no defects in T-cell development [27].

BTLA-deficient mice exhibit T-cell hyper-reactivity and increased susceptibility to autoimmune disorders compared to WT mice. Upon stimulation with anti-CD3 antibodies (Abs) BTLA-deficient T-cells proliferate more vigorously and produce higher amounts of proinflammatory cytokines after activation than WT T-cells [9]. Moreover, BTLA-deficient mice show an increased number of memory CD8 + T-cells, suggesting that CD8 + BTLA-deficient T-cells differentiate to a greater extent into memory CD8 + T-cells [31]. In another study, it was shown that BTLA cross-linking with agonistic BTLA monoclonal Abs (mAbs) resulted in reduced T-cell proliferation and cytokine production (IFNγ and IL-10) upon anti-CD3 stimulation [23]. It has been also noticed that T-cell hyper-reactivity in BTLA-deficient mice may be associated with an impaired tolerance or dysregulation of the Treg function [32]. In mouse models of airway hypersensitivity, the duration of lung inflammation was longer in BTLA-deficient mice suggesting that BTLA can act not only at the early stages of T-cell activation but additionally may regulate T-cell survival during T-cell-mediated inflammation. BTLA-deficient T-cells displayed decreased apoptosis and thus could mediate prolonged inflammation [33, 34]. Additionally, it has been demonstrated that BTLA contributes to the induction of peripheral tolerance of both CD4 + and CD8 + T-cells in mice [35].

### B-cells

While mature B-cells express the highest levels of BTLA among all lymphocytes, it’s worth noting that the majority of our knowledge about BTLA is derived from studies focused on T-cells. BTLA expression can be detected on B-cells from the early stages of B-cell differentiation in bone marrow. BTLA is detected at low levels in bone marrow-derived precursor B-cells and increases during pro-B and pre-B-cell maturation [27, 28, 36, 37]. B-cell development remains unaffected by BTLA, as normal B-cell development was observed in BTLA-deficient mice. In comparison to B-cells derived from the bone marrow or tonsil tissue, B-cells isolated from peripheral blood (PB) express high levels of BTLA [36]. Among mature B-cell subsets, peripheral naïve and resting B-cells constitutively express the highest levels of BTLA, while plasmablasts display lower BTLA levels compared to naïve B-cells. When compared to naïve and plasmablasts, all memory B-cell subtypes display the lowest BTLA levels [36, 38]. BTLA levels also depend on the activation status of B-cells, decreasing upon B-cell activation [25]. Moreover, BTLA expression on B-cells decreases with age, leading to a reduced response to the trivalent influenza vaccine and a reduced capacity for IgG Abs production [39].

When activated by HVEM, BTLA associates with the BCR signaling complex, however, BTLA was not found to have an impact on the BCR signaling complex activation itself [36]. BTLA attenuates BCR signaling by inhibiting the phosphorylation of the tyrosine kinase SYK protein, B-cell linker protein (BLNK), and phospholipase Cγ2 (PLCγ2). Moreover, BTLA activation results in the downregulation of NF-κB activation [36]. In this manner, BTLA-HVEM ligation suppresses co-stimulatory molecule upregulation leading to reduced activation of BCR downstream signaling molecules [36]. Furthermore, studies have documented that BTLA-HVEM signaling inhibits B-cell proliferation and secretion of specific cytokines (IL-6, IL-10, and TNFα), but not chemokines (IL-8 and MIP-1β) [40].

### Dendritic cells

In addition to B and T-cells, BTLA is also expressed in dendritic cells (DCs). BTLA is expressed at low levels on immature DCs and its level increases during DCs maturation [41]. BTLA is not essential for DC development since in BTLA-deficient mice the number of DCs was similar to WT mice [42]. However, BTLA overexpression can suppress the maturation of DCs and promote immune tolerance of immature DCs [43]. Additionally, the BTLA-HVEM pathway is involved in the regulation of DC homeostasis. BTLA-HVEM signaling suppresses the proliferation of DCs, indicating that the BTLA-HVEM pathway provides inhibitory signaling for DCs homeostasis in lymphoid tissue [44]. Furthermore, BTLA-deficient mice are more susceptible to LPS-induced endotoxic shock than WT mice and that LPS-induced BTLA-deficient DCs produced more TNFα and IL-12 [42]. Moreover, the induction of peripheral Treg cells relies on DEC205 + DC population expressing BTLA. BTLA + DCs promote Foxp3 expression in T-cells through the upregulation of CD5 leading to Treg cell differentiation and the induction of peripheral Treg cell tolerance [45]. It has been shown that active tuberculosis (TB) drives BTLA expression in DCs, leading to reduced expression of DC-maturation markers, decreased IL-12/IFNα production, and increased IL-4 and TGFβ production, and in consequence to suppression of Th17 and Th22 response while promoting Th2 and Foxp3 + Treg differentiation [46].

## 1BTLA in Cancer

Dysregulation of the BTLA/HVEM axis has been described in a plethora of solid tumors as well as in hematological malignancies. Abnormal BTLA expression, particularly within the tumor microenvironment (TME) and specifically on tumor-infiltrating lymphocytes (TILs), has been observed in diverse cancers and is frequently associated with impaired anti-tumor immune responses. Where upregulated BTLA expression has been linked to the inhibition of anticancer responses, increased frequency of BTLA + T-cells, and unfavorable disease outcomes. Nevertheless, despite numerous publications underscoring the significance of dysregulated BTLA expression in cancer, there remains a dearth of knowledge regarding the regulation of BTLA protein expression at the translational and post-translational levels. So far, it has been demonstrated that expression of serine/arginine-rich splicing factor 2 (SRSF2) in renal cell carcinoma patients (RCC) is involved in the regulation of IC expression (*inter alia* BTLA), which in turn modulates TILs’ exhaustion. Furthermore, SRSF2 was revealed to regulate the transcription of those immune checkpoint genes by associating with an acyl-transferases P300/CBP complex and altering the H3K27Ac level near these genes, thereafter influencing the recruitment of signal transducer and activator of transcription 3 (STAT3) to those gene promoters [47].

Regarding epigenetic, only miRNAs have been the subject of research concerning the BTLA regulation. The initial indication of miRNA-mediated regulation of BTLA expression was provided by Loeb et al. [48]. In their exploration of a transcriptome-wide miR-155 binding map, they demonstrated that both miR-155 and miR-17 can bind to the BTLA sequence through noncanonical motifs, consequently exerting a negative regulatory influence on B and T cell proliferation [48]. Supporting this data, subsequent research highlighted the significance of miR-155-5p in BTLA regulation [49, 50]. In a mice model, it was discovered that miR-155 plays a crucial role in governing BTLA expression during naïve CD4 + T cell activation [50]. Notably, our work contributes to this understanding, demonstrating that miR-155-5p plays a crucial role in regulating BTLA expression in human CLL cells [49]. Additionally, the study beyond miR-155, demonstrated that miR-32, one of the downregulated miRNAs in ovarian cancer, exerts inhibitory effects on the proliferation, migration, and invasion of ovarian cancer cells by targeting BTLA [51]. These findings underscore the intricate regulatory mechanisms involving miRNAs in modulating BTLA expression with potential implications for cancer.

### Hematological cancers

Shortly after the discovery of BTLA as a lymphoid receptor that inhibits lymphocyte activation, scientists started exploring BTLA expression and its potential role in malignancies of the lymphatic system. In one of the first studies on BTLA expression patterns, M’Hidi et al. described the distribution of BTLA in different human lymphomas using immunohistochemistry (IHC). Among B-cell non-Hodgkin lymphomas (NHL), only small lymphocytic lymphoma (B-SLL) exhibited a strong BTLA signal in all cases. Other B-cell NHLs, including mantle cell lymphoma (MCL), follicular lymphoma (FL), diffuse large B-cell lymphoma (DLBCL), marginal zone lymphoma (MZL), and Burkitt lymphoma (BL), showed no or a weak BTLA signal. In the case of T-cell NHL, all neoplastic T-cells of T-NHL were found to be BTLA-positive. In Hodgkin lymphomas (HL) no positive BTLA staining was detected on Reed-Sternberg cells. However, both in B-NHL and HL, BTLA signals were detected on reactive T-cells at various levels. Additionally, the highest levels of BTLA among B-NHL were present on chronic lymphocytic leukemia (CLL) neoplastic B-cells [52]. These findings were confirmed by Trougouboff et al. [53]. Furthermore, Liao et al. showed that T-cells from peripheral T-cell lymphoma (PTCL) patients express higher BTLA levels than healthy controls (HC), with the highest levels in advanced-stage PTCL patients [54].

In subsequent studies on BTLA expression patterns in lymphomas, Carreras et al. examined BTLA expression in lymph node biopsies using IHC. BTLA + cells were detected in the germinal center of the follicular area as well as in the interfollicular compartment, following a pattern of T-cell distribution. Moreover, in 36% of FL cases, BTLA + cells were also localized in the mantle and marginal zone area, following a B-cell staining pattern. In the germinal center, BTLA + Tfh cells co-localized with PD-1 + Tfh cells. Furthermore, in the follicular area, BTLA expression levels positively correlated with PD-1 and FOXP3 expression. High BTLA levels were associated with a favorable overall survival (OS). Moreover, in patients treated with rituximab, high BTLA levels correlated with a better prognosis [55].

In another study, Quan et al. examined BTLA expression and function of T-cells from the TME of DLBCL patients. The proportion of BTLA + CD3 + T-cells was higher in DLBCL than in HC. Furthermore, high BTLA levels correlated with advanced stages of DLBCL. BTLA-positive CD3 + T-cells co-expressed upregulated levels of PD-1, TIM-3, LAG-3, and LIGHT, compared to BTLA-negative CD3 + T-cells. Moreover, BTLA + CD3 + CD8 + T-cells, but not BTLA-CD3 + CD8 + T-cells, produced less perforin and granzyme B. In addition, BTLA + CD4+/CD8 + T-cells expressed higher levels of pSTAT3 than BTLA-CD4+/CD8 + T-cells. Therefore, TME BTLA + T-cells of DLBCL patients displayed a less differentiated phenotype, lower cytolytic function, and were more prone to proliferate in response to IL-2 than BTLA- T-cells [56].

In one of our studies, BTLA expression patterns were described in PB CD19 + B-cells and CD3 + T-cells of CLL patients. Compared to HC, BTLA mRNA expression levels were found to be highly upregulated in both CD19 + and CD3 + cells. At the protein level, BTLA expression, as well as the frequency of BTLA + CD19 + and BTLA + CD3 + cells, was lower in patients compared to HC. Moreover, BTLA + CD3 + cells showed a higher expression of both surface and intracellular CTLA-4 protein in patients compared to HC, while BTLA + CD19 + cells showed a lower expression of both intracellular and surface CTLA-4 protein in patients. BTLA levels were higher in CD3 + T-cells of patients than in HC. Furthermore, the number of BTLA + CTLA-4 + CD3 + T-cells was higher in the CLL group, while the frequency of BTLA + CTLA-4 + CD19 + B-cells was similar between groups. PMA stimulation decreased BTLA expression in HC and to a lesser extent in CLL, demonstrating that CLL BTLA + CD19 + and BTLA + CD3 + cells are less prone to in vitro stimulation [25]. In two other studies by Sordo-Bahamonde et al., the role of the BTLA-HVEM axis in the regulation of leukemic B-cells, T-cells, and NK-cells in CLL was investigated. On the basis of the GEO study, the authors confirmed higher BTLA mRNA expression in CLL. In contrast to our findings, Sordo-Bagamonde’s group documented increased BTLA protein levels on CLL B-cells compared to HC. Among T-cell subsets, BTLA expression was found to be upregulated on CD3+, CD4+, and CD8 + T-cells of CLL patients. Moreover, BTLA levels and the number of BTLA + NK-cells were increased in CLL. In addition, BTLA expression on CLL B-cells correlated with the frequency of BTLA + NK-cells. The Kaplan-Meier analysis did not reveal a correlation between BTLA mRNA expression in CLL B-cells and patient survival. However, high BTLA expression on NK-cells and CD4+, but not CD8 + T-cells, was associated with a shorter time to treatment (TTT). Functional studies showed that BTLA blockade increased the percentage of IFNγ + NK-cells and IFNγ + CD8 + T-cells, decreased the secretion of IL-10 by CLL B-cells, and increased tumor cell lysis. This effect was even stronger when anti-BTLA Abs were used together with bispecific anti-CD3/anti-CD19 Abs [57, 58].

### Lung cancer

Mittal et al. examined the impact of lung malignancy on the phenotype and function of CD8 + and CD4 + T-cells in a murine lung cancer model. They observed an increase in the percentage and absolute number of BTLA + CD8+/CD4 + T-cells in mice with cancer, along with other ICs, such as PD-1 and 2B4. Furthermore, BTLA + CD4 + T-cells produced less IL-2 compared to those from healthy mice, while BTLA + CD8 + T-cells in tumor- bearing mice had impaired IL-2 and TNF production. However, there was no difference in IFNγ production. These results suggest that lung tumor development leads to an enhanced expression of BTLA, PD-1, and 2B4 on CD8 + and CD4 + T-cells, resulting in reduced effector functions of tumor-specific T-cells [59].

Wang et al. studied the expression of BTLA in T-cells present in pleural fluid and PB from lung cancer patients. They observed no differences in BTLA expression on PB T-cells between patients and HC. However, T-cells from pleural fluid exhibited higher levels of BTLA compared to PB T-cells from the same patients. The authors suggest that upregulation of BTLA expression occurs on activated T-cells in the pleural fluid, and therefore the presence of BTLA + T-cells may negatively regulate the local immune response [60].

Thommen et al. investigated the expression pattern of inhibitory ICs on CD8 + TILs in non-small cell lung cancer (NSCLC). They found that PD-1 was the primary IC expressed on CD8 + TILs, whereas BTLA, TIM-3, CTLA-4, and LAG-3 were expressed at lower levels. In contrast, CD8 + T-cells from the PB of HC showed no expression of PD-1, TIM-3, CTLA-4, and LAG-3, while BTLA was mainly expressed on naïve T-cells. Although the frequency of BTLA + CD8 + T-cells was low in most cases, there was a slight, yet not statistically significant, increase in BTLA levels in advanced stages. When compared to PD-1 + CD8 + TILs, which on average expressed the lowest levels of other ICs, BTLA + CD8 + TILs exhibited high levels of these receptors, indicating that upregulated BTLA expression is a characteristic feature of late-stage T-cell exhaustion. Based on these observations, the authors proposed a sequential expression pattern of ICs, where PD-1 is expressed first, followed by TIM-3, CTLA-4, and LAG-3, and finally BTLA [61].

Using IHC, Li et al. studied BTLA expression and its correlation with PD-1 and PD-L1 presence on TILs and tumor tissue in NSCLC. They discovered that BTLA can be expressed on tumor cells, being primarily detected in the membrane and cytoplasm of cancer cells and occasionally on TILs. Additionally, BTLA levels were significantly higher in patients with lymph node metastasis and advanced disease. The Kaplan-Meier analysis revealed that BTLA-positive patients had a shorter recurrence-free survival (RFS) and OS compared to BTLA-negative patients. Furthermore, patients who were negative for both BTLA and PD-L1 had longer RFS and OS than patients who were double or single positive for these markers. These findings indicate that high levels of BTLA may serve as a predictor of disease progression and poor prognosis in NSCLC [62]. In contrast to Li’s findings, Shen et al. documented that BTLA can only be detected on TILs in IHC cancer sections [63]. This discrepancy may be attributed to the difference in Abs used. Li’s study employed a polyclonal BTLA Ab for IHC, while Shen’s study used a BTLA mAb.

Finally, based on the integrated analysis of two large datasets, the Cancer Genome Atlas (TCGA) and PROSPECT, Lou et al. investigated the correlation between the presence of the epithelial-mesenchymal transition (EMT) and the expression of ICs in lung adenocarcinoma (LUAD). They found that tumors with EMT exhibited upregulated expression of ICs, including BTLA, in the mesenchymal tissue of LUAD. This suggests that increased BTLA expression may modulate the tumor EMT through the inflammatory microenvironment [64]. In a similar analysis, Zhang et al. examined the prognostic value of complement-related genes in LUAD using data from TCGA. The authors classified patients into two different clusters and stratified the prognosis of LUAD into different risk categories using complement-related gene signatures. They found that an enriched infiltration of T cells, B lineage cells, myeloid dendritic cells, neutrophils, and endothelial cells was observed predominantly in the low-risk group with a better prognosis. Furthermore, they assessed the correlation between several prominent immunotherapeutic biomarkers and the complement-related risk score. The results showed that the expression level of BTLA was significantly associated with the prognosis of LUAD, with high levels present in the low-risk group [65].

### Melanoma

Derre et al. demonstrated that effector CD8 + T-cells specific to tumor antigens (TMA) in human melanoma, whether circulating or in solid tumors, consistently exhibited high levels of BTLA. Moreover, high BTLA expression was also observed in memory and effector T-cells, while progressive differentiation of total CD8 + T-cells into effector cells was associated with reduced BTLA expression [26]. In another study, Malissen et al. documented that 2.1–35.6% of CD8 + and 12.6–61.5% of CD4 + TILs from metastatic melanoma patients expressed BTLA on their surface. Furthermore, BTLA + CD8 + T-cells were found in close proximity to HVEM-positive melanoma cells. These findings suggest the involvement of the BTLA-HVEM axis in a melanoma’s evasion of T-cell surveillance [66].

Radvanyi et al. sought to identify predictive biomarkers of the adoptive cell therapy (ACT) effectiveness. They performed immunophenotyping of infused TILs and observed that the percentage and total number of CD8 + TILs were associated with treatment response. Further, the expression of PD-1, TIM-3, and BTLA on CD8 + TILs varied among patients. Responders had significantly higher frequencies of CD8 + BTLA + and CD8 + BTLA + TIM-3 + TILs [67]. In a subsequent study, Haymaker et al. aimed to characterize the CD8 + BTLA + TIL subset in relation to the ACT response. They found that CD8 + BTLA + TILs showed better responsiveness to IL-2 stimulation, exhibited less differentiation into memory-effector T-cells, and persisted longer in patients after TIL infusion compared to CD8 + BTLA- TILs. Upon activation by HVEM, BTLA-mediated signaling inhibited proliferation and cytokine production of CD8 + BTLA + TILs, but promoted their survival through activation of the Akt/PKB pathway. The authors suggested that the presence of CD8 + BTLA + TILs and their less differentiated state, as well as the survival abilities induced by BTLA signaling, correlated with a positive treatment outcome [68].

Fourcade et al. aimed to identify immune escape mechanisms responsible for the dysfunction of tumor-specific NY-ESO-1 CD8 + T-cells in patients with advanced melanoma. NY-ESO-1-specific CD8 + T-cells induced by tumors exhibited upregulated BTLA expression compared to virus-specific and total CD8 + T-cells. The majority of BTLA + NY-ESO-1-specific CD8 + T-cells also co-expressed elevated levels of PD-1, with PD-1 + BTLA + TILs being the most abundant subset among NY-ESO-1-specific CD8 + T-cells. Functional evaluation revealed that PD-1 + BTLA + cells are more dysfunctional than PD-1 + BTLA- cells and less dysfunctional than BTLA + PD-1 + TIM-3 + cells since the PD-1 + BTLA + cells produced less IFNγ, TNF, and IL-2 than PD-1 + BTLA- cells, and more than the BTLA + PD-1 + TIM-3 + cells. Additionally, in contrast to PD-1 and TIM-3, BTLA expression did not increase upon stimulation with tumor antigens on any NY-ESO-1-specific CD8 + subpopulation. Antigen BTLA blockade resulted in an increased capacity of NY-ESO-1-specific CD8 + T-cells to produce IFN-γ, TNF, and IL-2 as well as enhanced the proliferation and expansion of NY-ESO-1-specific CD8 + T-cells. Those effects were even stronger in combination with anti-PD-1 or anti-PD-1/anti-TIM-3 Abs [69].

To understand how melanoma cells influence IC expression, Gestermann et al. co-cultured PBMCs from HC with melanoma cell lines. The expression levels of PD-1, TIM-3, LAG-3, and BTLA were detected on CD8+/CD4 + T-cells at various levels. BTLA was found to be expressed at intermediate to high levels on primary CD8+/CD4 + T-cells, and its expression decreased during co-culture with melanoma cells in both T-cells populations. Furthermore, the effectiveness of PD-1, TIM-3, LAG-3, and BTLA blockade was tested. The presence of anti-BTLA Abs alone or in combination with other blocking Abs decreased melanoma cell numbers and increased the T-cell to melanoma cell ratio. Therefore, Gestermann’s study showed the potential application of the combined IC therapy, including anti-BTLA Abs, in the treatment of melanoma [70].

Ersek et al. explored the influence of melanoma-associated fibroblasts (MAFs) on IC expression in CD8 + T-cells. BTLA levels were analyzed on PBMCs from HC co-cultured with MAF-conditioned media. Under MAF stimulation, BTLA and TIGIT expression increased on CD45RO + non-naïve/memory cytotoxic T-cells. This effect was even stronger when exposed to MAFs with upregulated arginase expression. In contrast, blocking arginase activity had the opposite effect on BTLA and TIGIT expression. Therefore, the authors proposed that aberrant IC expression on CD8 + T-cells in the presence of soluble MAF factors is an arginase-mediated phenomenon [71].

Dong et al. used the TCGA and GTEx RNA-Seq data to evaluate BTLA expression and its clinical value in skin cutaneous melanoma (SKCM). BTLA mRNA expression was found to be higher in metastatic patients than in patients with primary melanoma and HC. Similarly, at the protein level, high BTLA levels correlated with metastatic SKCM. The analysis of immune cell infiltration levels in primary and metastatic SKCM revealed a significant association between BTLA levels and the type as well as the degree of abundance of specific immune cell types. In addition, survival analysis showed that the high BTLA group had longer OS, disease-specific survival (DSS), and disease-free interval (DFI) than the low BTLA group. Moreover, high BTLA patients were more likely to respond to melanoma-associated antigen A3 (MAGE-A3) blocker and anti-PD-1 treatment [72].

### Gastrointestinal cancers

Only a few studies have investigated the prognostic value of BTLA in gastrointestinal cancer. Feng et al. evaluated the expression and prognostic significance of BTLA in patients with stage IIIa gastric adenocarcinoma by IHC. They observed no BTLA staining in normal mucosal tissues, while BTLA-positive gastric cancer (GC) cells were found in 75.6% of patients. Among them, 41.9% displayed high BTLA levels which correlated with tumor location and shorter patient OS [73]. In a similar study, Lan et al. investigated BTLA and HVEM expression in GC. The BTLA-positive signal was mainly located within the cytoplasm of GC cells and detected in 74.3% of specimens. High BTLA levels were found in 37.5% of all BTLA-positive patients and associated with lymph node metastasis as well as negatively correlated with patient survival. Correlation analysis also showed a positive correlation between BTLA and HVEM expression. Moreover, elevated BTLA and HVEM levels were associated with poor prognostic factors [74]. In their recent study, Azarafza et al. analyzed BTLA mRNA and protein levels in GC. RT-qPCR results showed significantly higher BTLA mRNA expression in advanced stages (III and IV) compared to early stages. IHC confirmed results at the mRNA level, showing higher BTLA levels in advanced disease than in early-stage disease [75]. In all of these studies, polyclonal anti-BTLA Abs were used for IHC, therefore, additional research with mAbs needs to be undertaken to confirm the findings presented above.

In colorectal cancer (CRC), Song et al. aimed to evaluate the expression of BTLA and its clinical significance in CRC patients using TIMER, Oncomine, and TCGA databases. Expression analysis revealed lower BTLA mRNA levels in CRC tissue compared to adjacent tissue. High BTLA expression correlated with better OS, disease free interval, and progression-free interval. Interestingly, elevated BTLA levels were found to be associated with lymph node metastasis. Despite this finding, the Cox regression analysis identified BTLA as a favorable prognostic factor in CRC. Furthermore, BTLA levels were related to tumor-infiltrating (TI) immune cell levels. Among them, with the exception of monocytes, M0 macrophages, and activated mast cells, which infiltrated the tumor to a lesser extent in cases with high BTLA, most tumor-infiltrating immune cells were found in greater numbers in the high BTLA group compared to the low BTLA group. Additionally, BTLA expression was associated with the expression of T-cell markers, B-cell markers, and NK-cell markers [76]. IL-21 expression was also found to be linked with BTLA levels, which is consistent with an observation by Kashiwakuma’s group that BTLA suppresses IL-21 production by Tfh cells to inhibit IgG production [77]. Functional analysis revealed the potential role of BTLA in the regulation of TCR and BCR signaling and NK-cell-mediated cytotoxicity pathways of TILs in CRC [76].

In another study, Kamal et al. investigated the expression levels of CTLA-4, TIM-3, LAG-3, and BTLA in PBMCs isolated from primary and treatment-naïve CRC patients using RT-qPCR. In contrast to Song et al., expression levels of all studied ICs, including BTLA, appeared to be upregulated in CRC. Correlation analysis showed a positive correlation of CTLA-4 expression with TIM-3, LAG-3, and BTLA expression. Additionally, the expression of TIM-3 strongly correlated with LAG-3 and BTLA levels. Finally, LAG-3 levels also positively correlated with BTLA expression. Moreover, patients with advanced disease showed significantly higher expression of those ICs than patients in the early stage, who in turn showed higher levels than HC. The Kaplan-Meier analysis demonstrated a negative correlation of CTLA-4, TIM-3, LAG-3, or BTLA expression with 1-year OS [78]. However, confirmation at the protein level, as well as a longer observation period than 1 year, is needed to confirm these findings.

Sorrentino et al. performed an analysis of the RNA-seq data from the TCGA database, as well as IHC in CRC patients, to identify markers of immune exhaustion associated with CRC and their relationships with patient survival. BTLA mRNA levels were found to be associated with worse patient outcomes. Similarly, to Kamal et al., the high BTLA group had a shorter OS compared to the low BTLA group. Furthermore, BTLA expression correlated with FCRL4, SIGLEC6, SIGLEC2 levels, and co-occurred with typical B-cell and T-cell markers. Therefore, the results obtained suggest the involvement of BTLA in the exhaustion of both B-cells and T-cells in CRC [79].

Oguro et al. performed IHC together with immunofluorescence (IF) staining to describe TI immune cells expressing BTLA and/or Cbl-b in gallbladder cancer (GBC). Control cases of xanthogranulomatous cholecystitis (XGC) and chronic cholecystitis (CC) were used in this study. BTLA was found to be expressed in CD3+, CD4+, CD8+, and CD20 + TILs, CD14 + monocytes, CD68 + macrophages, CD1a+, CD207+, and CD208 + DCs. However, FOXP3 + cells, CD56 + NK-cells, or Cbl-b + cells did not express BTLA in tumor tissue. Moreover, BTLA and Cbl-b were exclusively expressed on TI immune cells and not on cancer cells. When the density ratios of a specific immune cell population were taken into account, FOXP3/CD4, BTLA/CD8, and Cbl-b/CD8 ratios were found to be higher in GBC compared to XGC and CC groups. Additionally, a high BTLA/CD8 ratio significantly correlated with venous invasion, perineural invasion, and histopathological grading. Survival analysis showed that a lower density of CD8 + TILs and a higher BTLA/CD8 ratio were associated with worse outcomes [80].

### Hepatocellular carcinoma

The expression pattern of BTLA in hepatocellular carcinoma (HCC) has been investigated among others by Zhao et al., who found that BTLA was significantly overexpressed on CD4 + T-cells in tumor tissue compared to adjacent tissue. Interestingly, over 85% of BTLA + CD4 + TILs co-expressed PD-1, while PD-1 + BTLA + CD4 + TILs accounted for 50% of all PD-1 + T-cells in HCC. Moreover, the frequency of PD-1 + BTLA + CD4 + TILs was associated with advanced HCC. Functional studies revealed that both tumor and non-tumor BTLA + CD4 + T-cells exhibited reduced capacity for IFNγ production compared to BTLA-CD4 + T-cells. Importantly, PD-L1 blockade restored the ability of IFNγ production in BTLA + PD-1 + CD4 + T-cells but partially suppressed the activation of BTLA-PD-1 + CD4 + T-cells [81]. Additionally, Liu et al. demonstrated that BTLA expression was upregulated on PB CD4 + T-cells but not on PB CD8 + T-cells in HCC. In contrast, the levels of HVEM expression were significantly downregulated on circulating CD8 + but not on CD4 + T cells. Both CD4 + and CD8 + T-cells in HCC displayed a reduced capacity for IFNγ production, and BTLA blockade restored IFNγ production, suggesting the potential role of BTLA in suppressing effector functions of T-cells in HCC [82]. In patients with hepatitis B virus-related HCC, Yi et al. observed higher BTLA expression levels on CD8 + effector memory (CCR7-CD45RA-) and CD45RA + effector memory (CCR7-CD45RA+) TILs compared to paired non-tumor and PB T-cells [83].

### Oral cancer

Yu et al. aimed to identify ICs that are positively correlated with PD-L1 expression and determine immune cell subpopulations in oral and squamous cell carcinoma (OSCC) based on data from TCGA and GEO databases. They found that eight ICs, including BTLA, were positively correlated with PD-L1 expression in OSCC. Except for ADORA2A, all of them were also negatively correlated with the tumor mutation burden (TMB). Survival analysis showed that most ICs, including BTLA, were significantly associated with OS. Combinations such as low PD-L1/ low BTLA and high CD8alpha/ low BTLA were predictive of a favorable prognosis in OSCC [84]. Similarly, Weber et al. performed a comparative analysis of IC expression in OSCC. Compared to healthy oral mucosa (NOM) tissue, levels of BTLA and PD-1 tended to be increased over 2-fold in OSCC, but without statistical significance. When IC expression in relation to CD8 levels was analyzed, BTLA and PD-1 showed over 4-fold higher expression in OSCC compared to NOM, but without statistical significance. Additionally, similar to Yu’s study, BTLA expression was not correlated with clinical parameters [85].

### Thyroid cancer

Qian et al. conducted a study on the characteristics of Tfh cells in patients with differentiated thyroid carcinoma (DTC). They found that in patients with distant metastasis, the frequency of CD4 + CXCR5 + Tfh-like cells was significantly higher compared to HC and patients without distant metastasis. Furthermore, BTLA was over expressed on Tfh cells in patients with cervical and distant metastasis compared to HC. Despite higher levels of BTLA and the increased proportion of Tfh cells in patients with metastasis, there was no significant difference in the frequency of BTLA + Tfh cells between metastatic patients and patients with localized tumors [86]. In another study, Luo et al. examined the expression pattern of ICs in thyroid carcinoma (TC) and its correlation with clinical parameters. IHC results indicated that similar to other ICs, BTLA was exclusively expressed on TILs in TC. However, there was no association between BTLA levels and clinical characteristics or patient survival [87].

### Gynecologic cancers

Imai et al. investigated the expression status of ICs in the TME of epithelial ovarian cancer (EOC) by flow cytometry. On average, 37.6% of CD4 + and 15.7% of CD8 + TILs expressed BTLA. However, no correlation was found between BTLA levels on CD4+/CD8 + TILs and clinicopathological features or patient survival [88]. Furthermore, Chen et al. evaluated the transcriptional level of BTLA in EOC tumor tissue. BTLA expression was detected in 59% of patients. BTLA-positive patients had higher incidences of advanced disease, disease relapse, and disease-related death compared to the BTLA-negative group. In contrast to Imai’s findings, Chen’s group found a correlation between detectable BTLA expression and worse OS [5].

Considering the presence of BTLA expression in the TME of EOC, Chen et al. evaluated the effectiveness of chemotherapy combined with BTLA blockade in EOC treatment. The survival of EOC mice treated with chemotherapy and anti-BTLA Abs improved significantly compared to chemotherapy or anti-BTLA alone. These mice showed higher frequencies of activated CD4 + and CD8 + splenocytes, as well as an increased release of pro-inflammatory cytokines such as IL-12, TNFα, and IFNγ. However, there were no differences in the levels of anti-inflammatory cytokines such as IL-6, IL-10, or TGF-β among different treatments. Flow cytometry data showed that BTLA was predominantly expressed on CD19 + B-cells, and its levels increased with disease progression along with increasing levels of IL-6, IL-10, or TGF-β. Functional studies revealed that IL-6 and IL-10, but not TGF-β, could induce BTLA expression in CD19 + B-cells, possibly through the activation of AKT/STAT3 signaling. The findings of the authors suggest that combined chemotherapy with BTLA blockade enhances immune activation and generates potent anti-tumor effects [5].

In the context of breast cancer (BC), Muenst et al. analyzed the expression of PD-1 and BTLA in tumor samples using IHC. PD-1 + TILs were detected in 15.8% of cases. Conversely, BTLA + TILs were observed in only 2.3% of cases, and all BTLA + cases were also PD-1+. Among all cells from freshly dissected tumors, 3.9% were PD-1+, and BTLA was expressed in less than 1% of all cells. Based on the observation that BTLA is rarely detected in BC cases, the authors concluded that BTLA does not play a biologically relevant role in BC immunosurveillance [89].

Fang et al., who used the UALCAN database to compare the expression levels of 50 ICs at the transcriptional level in BC, noted that BTLA mRNA expression is down-regulated in BC. However, variations in BTLA expression did not show any correlation with either clinicopathological characteristics or patient survival [90]. Similarly, Filippi et al. analyzed transcriptomics data of 742 genes from the TCGA database and identified 6 IC genes, including BTLA, to be expressed at lower levels in BC patients, while in contrast to Fang et al., low BTLA expression was associated with poor disease prognosis [91].

Sekar et al. investigated the expression levels of PD-1 and BTLA on type I NKT-cells in a murine autochthonous mammary tumor model driven by the polyoma middle T oncogene (PyMT), as well as the consequences of BTLA blockade on tumor progression and metastasis. Type I NKT-cells represented a minor population of TILs, whereas conventional T-cells were very abundant in isolated tumors. Expression levels of PD-1 were comparable between the T-cell populations studied, while abundant BTLA expression was detected only on type I NKT-cells. Inhibition of BTLA significantly decreased tumor growth and tended to reduce pulmonary metastasis but did not prevent the outgrowth of new tumors. In those mice, BTLA blockade led to the increase in NK1.1 + T-cells and CD1d tetramer-recognizing type I NKT-cells loaded with PBS-57. Moreover, the expression levels of perforin and granzyme B increased under anti-BTLA treatment. Therefore, the authors suggest that inhibition of BTLA potentially contributes to the expansion of NKT-cell numbers, and the frequency of other T-cells with cytotoxic potential, resulting in reduced tumor growth. Additionally, Sekar’s group explored the METABRIC dataset to analyze BTLA expression in patients with mammary cancer. No correlation was found between BTLA levels and patient survival. However, when BTLA expression was analyzed together with the *ZBTB16* gene expression, a lineage-determining transcription factor of NKT-cells, the high ZBTB16/high BTLA group had significantly worse OS compared to the high ZBTB16/ low BTLA group [92].

### Neurological cancers

Woroniecka et al. aimed to describe T-cell exhaustion signatures in glioblastoma (GBM). They measured the expression of ICs on TILs and PBMCs in GBM. BTLA was found to be expressed at lower levels on TILs compared to PBMCs from both GBM patients and HC. In the case of PBMCs, there were no significant differences in the levels of ICs between patients and HC [93]. In a similar study, Shen et al. investigated the expression of ICs in the human glioma microenvironment (GME) and PBMCs isolated from high-grade glioma (HGG) patients. Flow cytometry results showed that myeloid cells, CD3 + CD4 + and CD3 + CD8-CD4- TILs expressed higher levels of BTLA compared to immune cells from healthy brain samples. Additionally, BTLA and LAG-3 appeared to be the most widely expressed ICs on TILs in GME. Furthermore, similarly to TILs, PBMCs from these patients displayed higher levels of BTLA, LAG-3, and TIM-3 compared to HC [94].

GBM has shown resistance to monotherapy with anti-PD-1 Abs, possibly due to the upregulation of multiple other ICs [95, 96]. Choi et al. evaluated the effectiveness of combination therapy using Abs against BTLA and PD-1 in GBM treatment in a murine GBM model. They found that BTLA expression significantly increased on non-Treg CD4 + TILs over time after GBM implantation in mice. Implementing anti-PD-1 and anti-BTLA combination therapy resulted in an increased expression and secretion of IFN-γ by CD4+/CD8 + TILs compared to non-treated control, as well as anti-PD-1 and anti-BTLA monotherapies. Moreover, a decrease in the proportion of CD4 + FoxP3 + Tregs in the brain was observed compared to monotherapy and control group. Further, GBM mice treated with combination therapy had a better OS than mice treated with anti-PD-1 monotherapy. However, in the anti-BTLA monotherapy group, there were no long-term survivors, and those mice had a similar OS as controls, showing no beneficial effect of anti-BTLA monotherapy on mice survival. In the case of mice depleted of CD4 + or CD8 + T-cells, there were no differences among the groups studied in terms of OS, suggesting that anti-PD-1 and anti-BTLA combination therapy provided a synergistic survival benefit in murine GBM, potentially through the reversal of immunosuppression of CD4+/CD8 + TILs [97].

Consistent with the results presented above, in a recent study by Ijang et al., the authors analyzed mRNA expression of BTLA in 33 different cancers using the TGAA database, as well as the prognostic value of BTLA on OS. Those findings confirmed the diverse consequences of BTLA expression in different cancers [98]. To summarize, the studies described above have been presented in Table 1. As shown, varied results have been observed in different cancers concerning BTLA mRNA expression in tumor and non-tumor tissue, as well as protein expression on tumor cells and TILs and its prognostic value. Furthermore, the data regarding response to BTLA blockade has shown a variety of functional consequences of such treatment. Those results highlight the significance of BTLA in cancer, shedding light on its potential as a therapeutic target and prognostic marker.

**Table 1:** Summary of studies on BTLA expression and function in cancer

Group[ref]	Cancer	Population	Main Method	BTLA expression	BTLA function	Correlation with OS
Liao et al. [54]	PTCL	Chinese	RT-qPCR	↑ mRNA in T-cells; ↑ in patients with advanced disease	-	-
Carreras et al. [55]	FL	Western FL patients	IHC	Detected in the germinal center of the follicular area as well as in the interfollicular compartment; In some cases, detected also in the mantle and marginal zone area	-	Yes; positive correlation
Quan et al. [56]	DLBCL	Chinese	FC	↑ % of BTLA + CD3 + T-cells; ↑ levels in patients with advanced disease	BTLA + CD8 + T-cells produced ↓ perforin and granzyme B; BTLA + CD4+/CD8 + T-cells expressed ↑ levels of pSTAT3	-
Karabon et al. [25]	CLL	Polish	FC	↑ mRNA levels in B and T-cells; ↓ on CLL B-cells	BTLA + CD19 + and CD3 + less responsive to PMA stimulation	-
Sordo-Bahamonde et al. [57, 58]	CLL	Spanish	FC	↑ on CLL B-cells, T-cells, NK-cells	BTLA activation led to↓IL-2 and IFNγ production; BTLA blockade resulted in ↑ IFNγ production and NK-cell/ CD8 + T-cell mediated response	Yes; positive correlation
Mittal et al. [59]	murine model of LC	-	FC	↑ on proportion of BTLA + CD8+/CD4 + T-cells	BTLA + CD8 + T-cells produced ↓ IL-2 and TNF	-
Wang et al. [60]	LC	Chinese	FC	↑ on pleural fluid T-cells in lung cancer patients	-	-
Thommen et al. [61]	NSCLC	Swiss	FC	Expressed on CD8 + TILs, but not on PB CD8 + T-cells	BTLA + CD8 + TIL co-expressed high levels of PD-1, TIM-3, CTLA-4, and LAG-3;	-
Li et al. [62]	NSCLC	Chinese	IHC	Expressed on tumor cells; ↑ in patients with lymph node metastasis and advanced disease	-	Yes; negative correlation
Shen et al. [63]	LUAD	Chinese	IHC	Expressed on TILs	-	-
Lou et al. [64]	LUAD	-	RS	↑ mRNA levels in patients with the present EMT	-	-
Zhang et al. [65]	LUAD	-	RS	↑ mRNA levels in low-risk group	-	-
Derré et al. [26]	melanoma	Swedes	FC	CD8 + T-cells expressed ↑ BTLA levels; Differentiation of CD8 + T-cells into effector cells was associated with ↓ BTLA expression	-	-
Malissen et al. [66]	melanoma	French	FC, IHC	Expressed on CD4 + and CD8 + TILs in contiguity with HVEM + melanoma cells	-	-
Radvanyi et al. [67]	melanoma	American	FC	↑ frequencies of CD8 + BTLA + and CD8 + BTLA + TIM-3 + TILs in responders to ACT	-	-
Haymaker et al. [68]	melanoma	American	FC	↑ on CD8 + TILs in patients responding to ACT therapy	↓ CD8 + BTLA + TILs proliferation and cytokine production; ↑ CD8 + BTLA + TILs survival	-
Fourcade et al. [69]	melanoma	American	FC	↑ on tumor-specific NY-ESO-1 CD8 + T-cell	BTLA blockade ↑ IFN-γ, TNF, and IL-2 production and proliferation by tumor-specific NY-ESO-1 CD8 + IFNγ + T-cell	-
Gestermann et al. [70]	melanoma	-	-	Expressed on CD4 + and CD8 + TILs	BTLA blockade led to ↓ melanoma cell number and ↑ T-cell:melanoma cell ratio	-
Érsek et al. [71]	melanoma	Hungarians	FC	Under MAF stimulation, BTLA expression ↑ on CD45RO + non-naïve/memory cytotoxic T-cells	-	-
Dong et al. [72]	melanoma	-	RS	↑ mRNA and protein levels in patients with metastatic	-	Yes, positive correlation
Feng et al. [73]	GC	Chinese	IHC	Positive staining in gastric cancer cells	-	Yes, negative correlation
Lan et al. [74]	GC	Chinese	IHC	Positive staining mainly within the cytoplasm of gastric cancer cells; ↑ levels associated with lymph node metastasis	-	Yes, negative correlation
Azarafza et al. [75]	GC	Iranian	RT-qPCR IHC	↑ mRNA and protein expression in advanced stages	-	-
Song et al. [76]	CRC	-	RS	↓ mRNA levels in cancer tissue; ↑ levels associated with lymph node metastasis; Associated with the degree of tumor infiltration and expression of B, T, and NK-cells markers	Potential role in the regulation of TCR and BCR signaling pathways and NK-cell-mediated cytotoxicity pathways of TILs	Yes, positive correlation
Kamal et al. [78]	CRC	Egyptian	RT-qPCR	↑ mRNA in PBMCs; ↑ in advanced stages; Correlation with CTLA-4, LAG-3, and TIM-3 levels	-	Yes; negative correlation
Sorrentino et al. [79]	CRC	-	RS	Correlation with FCRL4, SIGLEC6, and SIGLEC2 levels as well as with B and T-cell markers	-	Yes; negative correlation
Oguro et al. [80]	GBC	Japanese	IHC, IF	Expressed in the proportion of CD3+, CD4+, CD8+, and CD20 + TILs, CD14 + monocytes, CD68 + macrophages, CD1a+, CD207+, and CD208 + DCs	-	Yes; negative correlation
Zhao et al. [81]	HCC	Chinese	FC	↑ on CD4 + T-cells; ↑ % of PD-1 + BTLA + CD4 + TILs associated with advanced stages	↓ capacity for IFNγ production by BTLA + CD4 + T-cells	-
Liu et al. [82]	HCC	Chinese	FC	↑ on PB CD4 + T-cells	↓ capacity to IFNγ production; BTLA blockade ↑ production of IFNγ in PB CD4 + and CD8 + T-cells	-
Yi et al. [83]	HCC	Chinese	IHC	↑ on CD8 + effector memory TILs	-	-
Yu et al. [84]	OSCC	-	RS	Positively correlated with PD-L1 expression; Negatively correlated with the TMB	-	Yes; negative correlation
Weber at al [85].	OSCC	German	NS	↑ in tumor samples	-	-
Qian et al. [86]	TC	Chinese	FC	↑ on Tfh	-	-
Luo et al. [87]	TC	Chinese	IHC	Expressed on TILs	-	No correlation
Imai et al. [88]	EOC	Japanese	FC	Expressed on 37.6% of CD4 + TILs and 15.7% of CD8 + TILs; Correlation between BTLA levels on CD4+/CD8 + TILs and age, stage, or histological type of EOC	-	-
Chen et al. [5]	EOC	Taiwan	RT-qPCR	Detected in 59% of patients; BTLA + had ↑ incidences of advanced disease	-	Yes; negative correlation
Muenst et al. [89]	BC	Swiss	IHC, FC	Detected in 2.3% of cases	-	-
Fang et al. [90]	BC	-	RS	↓ mRNA expression	-	-
Filippi et al. [91]	BC	-	RS	↓ mRNA expression	-	Yes; negative correlation
Sekar et al. [92]	murine model of MC	-	FC, RS	Detected on type I NKT-cells	BTLA blockade led to ↓ tumor growth and to ↑ of NK1.1 + T-cells and PBS-57-loaded CD1d tetramer-recognizing type I NKT-cells numbers, and to ↑ production of perforin and granzyme B	No correlation
Woroniecka et al. [93]	GBM	Polish	FC	↓ on TILs	-	-
Shen et al. [94]	HGG	Chinese	FC	↑ on CD3 + CD4 + and CD3 + CD8-CD4- TILs; ↑ on PBMC	-	-
Choi et al. [97]	murine model of GBM	-	FC	↑ on TILs non-Treg CD4 + T-cells over time after tumor implantation	anti-PD-1 and anti-BTLA combination therapy resulted in ↑ IFN-γ expression and secretion by CD4 + and CD8 + TILs	-

HC– healthy controls, FC - flow cytometry, IHC - immunohistochemistry, RS - RNA-seq data, NS -NanoString, EMT - epithelial-mesenchymal transition, TMB - tumor mutation burden, LC - lung cancer, GC - gastric cancer, GBC - gallbladder cancer, HCC - hepatocellular carcinoma, OSCC - oral and squamous cell carcinoma, EOC - epithelial ovarian cancer, TC - thyroid carcinoma, BC - breast cancer, MC - mammary cancer, GBM - glioblastoma, HGG - high-grade glioma, ACT - adoptive cell therapy

## sBTLA and cancer

The first evidence of the existence of sBTLA in humans comes from Wang et al., who developed an ELISA test for the detection of sBTLA in serum. Their study demonstrated for the first time that sBTLA is present in serum of HC. Moreover, Wang’s group noticed that the concentration of sBTLA increases with age, prompting them to conclude that sBTLA may correlate with age-related immune dysregulation [99]. This observation suggests a key role for sBTLA in immunoregulation. Based on this discovery in recent years, more and more papers have appeared describing the predictive value of sBTLA in various diseases [100–102].

One of the first studies on the clinical significance of sBTLA in cancer comes from Wang et al., who studied the predictive value of soluble IC-related proteins in clear cell RCC (ccRCC). High sBTLA and sTIM-3 levels were associated with a decreased survival time in ccRCC [103]. Similarly, to Wang’s group, Bian et al. observed a negative correlation between plasma levels of sBTLA, sBTN3A1, pan-sBTN3A, sPD-1, and sPD-L1, and survival of patients with pancreatic adenocarcinoma (PDAC) [104]. The study by Dong et al. also suggests that sBTLA could be a prognostic marker for OS in advanced HCC since the low BTLA group lived longer than the high BTLA group [105]. Similarly, a high concentration of sBTLA in plasma was associated with the decreased survival time of patients with advanced high-grade serous ovarian cancer [106]. Wang et al. evaluated the role of serum ICs levels in newly diagnosed prostate cancer (PCa) patients with localized disease. sBTLA and sHVEM were identified as predictors of PCa progression. Moreover, sBTLA levels appeared to be a significant predictor of progression-free survival (PFS), where high levels of sBTLA correlated with more aggressive PCa [107]. In the same way, Sordo-Bahamonde et al. showed that sBTLA can be a prognostic marker in myeloid malignancies. Compared to HC sBTLA, serum levels were elevated in CLL patients and high sBTLA levels were associated with reduced OS in those patients. Additionally, increased levels of sBTLA have been found to be associated with poor prognostic markers like unfavorable cytogenetic alterations and a shorter time to treatment in CLL [57]. In another study, Gorgulho et al. confirmed that in patients with different solid malignancies, sBTLA serum levels were significantly elevated compared to HC. Moreover, sBTLA serum levels proved to be an independent prognostic factor for OS where high sBTLA concentration negatively correlated with patient OS. It is noteworthy that sBTLA levels exhibited a correlation with the frequency of PB CD3 + CD8 + BTLA + T-cells. When clinical characteristics such as age, gender and smoking status were taken into account, the baseline level of sBTLA was comparable between patients with different tumor entities. However, patients with UICC stage IV tumors had higher sBTLA serum levels compared to stage III patients [108]. Finally, Świderska et al. aimed to evaluate the clinical significance of sBTLA as a diagnostic and prognostic marker in patients with EOC. Serum levels sBTLA were found to be significantly higher in patients compare to HC. Moreover, sBTLA levels in serum and in peritoneal fluid correlated positively with grade score. Notably, elevated sBTLA levels in peritoneal fluid were found to influence OS [109]. On the contrary, Wang et al. evaluating the association of soluble ICs with the risk of NSCLC found no differences in sBTLA levels between NSCLC patients and HC [110]. Consistent with Wang et al., Peng et al. showed that sBTLA was rarely expressed in serum samples from NSCLC patients [111]. Similarly, Botticelli et al. demonstrated in advanced head and neck cancer (HNCa) that, contrary to sBTLA, it was the levels of sLAG-3 that exhibited an association with OS [112].

Furthermore, sBTLA concentration has also been investigated as a predictive biomarker for the treatment outcome in different types of cancer. The abovementioned study by Dong et al. suggests that sBTLA may be a useful biomarker for predicting OS in patients with advanced HCC treated with sorafenib and similar to results achieved in the overall analysis, those HCC patients who had high levels of sBTLA had a shorter OS after treatment than those with low levels of sBTLA [105]. Also, Gorgulho et al. evaluated the role of sBTLA as a predictive biomarker of the IC blockade response in solid malignancies. This study demonstrated that sBTLA concentration appeared to have the potential to serve as an IC blockade outcome prediction factor [108]. In contrast, Billon et al. did not document any impact of the plasma sBTLA level on patient survival with metastatic RCC treated with the IC blockade. Thus, sBTLA has not been shown to be a good predictive biomarker for nivolumab therapy in metastatic RCC patients previously treated for metastatic RCC with TKI [113]. Similarly, Botticelli et al. found that sLAG, while not sBTLA, can serve as a predictive biomarker in advanced HNCa patients treated with chemo- or immune-therapy [112].

Collectively, these studies suggest that the soluble form of the BTLA protein present in serum may serve as a prognostic factor of disease progression and as a predictive marker in response to treatment in some types of cancer. The role of sBTLA in cancer pathogenesis has not been established yet. Therefore, further studies on the function and role of sBTLA in cancer pathogenesis and progression are needed. A summary of the studies on sBTLA in cancer is presented in Table 2.

**Table 2:** Summary of studies on sBTLA in cancer

Group [Ref]	Cancertype	sBTLA in cancer	Correlation with OS
Wang et al. [103]	ccRCC	-	Yes; negative correlation
Bian et al. [104]	PDCA	-	Yes; negative correlation Patients with high level of sBTLA (> 1.91 ng/ml) had shorter OS (3.4 vs. 17.4 months)
Dong et al. [105]	HCC	-	Yes; negative correlation Patients with high level of sBTLA (> 395 pg/ml) had shorter OS (8.4 vs. 20.3 months)
Fanale et al. [106]	OC	-	Yes; negative correlation Patients with high level of sBTLA (> 2.78 ng/ml) had shorter OS (24 vs. 32 months)
Wang et al. [107]	PCa	sBTLA levels were associated with cancer aggressiveness	Yes; negative correlation High sBTLA levels correlated with shorter time to progression
Sordo-Bahamonde et al. [57]	CLL	↑ sBTLA levels in sera compared to HC; ↑ levels of sBTLA in the advanced Rai vs. 0 stage and Binet C stage; ↑ levels of sBTLA in patients with cytogenetic alterations	Yes; negative correlation
Gorgulho et al. [108]	solid malignancies	↑ sBTLA levels compared to HC; Correlation of sBTLA levels with the frequency of peripheral blood CD3 + CD8 + BTLA + T-cells; ↑ sBTLA levels in patients with the UICC stage IV than stage III	Yes; negative correlation Patients with high level of sBTLA (311.64 pg/mL) had shorter OS (138 vs. 526 days)
Świderska et al. [109]	EOC	↑ serum and peritoneal fluid sBTLA levels compared to HC	Yes; negative correlation
Wang et al. [110]	NSCLC	No association found	No correlation
Peng et al. [111]	NSCLC	sBTLA was rarely expressed in serum	-
Botticelli et al. [112]	HNCa	No correlation with clinical features found	No correlation

HC-healthy controls, ccRCC - clear cell renal cell carcinoma, PDAC - pancreatic adenocarcinoma, HCC - advanced hepatocellular carcinoma, OC - advanced high-grade serous ovarian cancer, PCa - prostate cancer, CLL - chronic lymphocytic leukemia, EOC - epithelial ovarian cancer, NSCLC - non-small cell lung carcinoma, HNCa - advanced high-grade serous ovarian cancer

## *BTLA* gene variants and cancer

Single nucleotide polymorphisms (SNPs) are the most common type of genetic variation in the human genome. SNPs located in gene sequences may affect their expression through various mechanisms and thus be associated with genetic susceptibility to various diseases, including cancer. Several previous studies have linked SNPs within the *BTLA* gene to the risk of cancer development and progression [114]. The characteristics and location of the SNPs studied in the *BTLA* gene are presented in Table 3; Fig. 1. A summary of the studies on the role of *BTLA* SNPs in cancer is presented in Table 4.

**Table 3:** Characteristics of *BTLA* gene single nucleotide polymorphisms

SNP	Location [GRCh38.p13]	Variation	Consequence
rs2633580	chr3:112500906	C > G	2KB Upstream Variant
rs11921669	chr3:112500581	C > T	2KB Upstream Variant
rs2633582	chr3:112500493	A > C	2KB Upstream Variant
rs2705565	chr3:112500489	C > T	2KB Upstream Variant
rs1844089	chr3:112498887	G > A	Intron Variant
rs2705535	chr3:112490080	C > T	Intron Variant - Potential role in splicing [115]
rs9288953	chr3:112484405	C > T	Intron Variant - Predicted to activate six new splice sites in splicing enhancer motifs and break one splicing sites in silencer motifs [116]
rs16859633	chr3:112479488	T > C; I(Ile) > V(Val)	Missense Variant Exon 2
rs2633562	chr3:112477584	T > C	Intron Variant
rs16859629	chr3:112471533	T > C	Intron Variant
rs2931761	chr3:112471290	G > T; R(Arg) > S(Ser)	Missense Variant Exon 3
rs76844316	chr3:112469762	T > G; N(Asn) > T(Thr)	Missense Variant Exon 4 - associated with decreased inhibitory activity of BTLA [117]
rs9288952	chr3:112466178	C > T; P(Pro) > L(Leu)	Missense Variant Exon 5
rs2171513	chr3:112466080	A > G	3’UTR Variant
rs1982809	chr3:112463893	A > G	3’UTR Variant - ↓ mRNA expression in T-cells of the CLL patients [118]
rs2705511	chr3:112460632	A > C	Intragenic region between CD200 and BTLA genes [-97,820 bp||-3334 bp]

### Breast cancer

One of the first studies on the association of *BTLA* polymorphisms with the risk of cancer came from Fu et al. Five *BTLA* SNPs; rs1844089, rs9288952, rs2633562, rs2705535, and rs2931761, were investigated in 592 Chinese women with malignant BC, and 506 age- and sex-matched HC. Fu’s group found that the frequencies of rs2705535 AG and rs1844089 CT genotypes were higher and increased BC risk by 1.5 and 1.3 times, respectively. Moreover, rs2705535 GG, rs1844089 CC, and rs9288952 CC genotypes were less frequent compared to HC and decreased BC risk by 1.4, 1.3, and 1.7 times, respectively. Haplotype analysis revealed correlation of the CAAAT (rs9288952, rs2931761, rs2633562, rs2705535. rs1844089) haplotype with a 3 times higher susceptibility to BC. Analysis including certain clinical factors showed correlation between *BTLA* SNPs and tumor size (rs1844089), estrogen receptor (ER) status (rs1844089, rs2633562, rs9288952), progesterone receptor (PR) status (rs2705535, rs9288952), C-erbB-2 status (rs2705535), and P53 status (rs1844089, rs2633562). Additionally, in patients ER-positive, PR-positive, P53-positive, and with lymph node metastasis the following haplotypes were more frequent: CAGAT, CAGAT, CAAGT/CAAAT, and TAAGT, respectively [119]. In another study, Zhao et al. investigated the effects of rs1982809 on the risk of BC in 324 Chinese women and 412 HC. The rs1982809 AA genotype was more prevalent in BC and increased BC risk by 2 times (AA vs. GG). Subgroup analysis revealed that the presence of rs1982809 increased BC risk in premenopausal women and women under the age of 55 years old. Additionally, rs1982809 correlated with clinicopathological features of BC, including C-erbB-2 status, Ki-67 status, ER status, TNM stage and tumor size [120].

### Chronic lymphocytic leukemia

Among hematological malignancies, the role of *BTLA* polymorphisms in CLL has only been investigated in our study by Karabon et al. The association between ten *BTLA* SNPs: rs2705511, rs1982809, rs9288952, rs76844316, rs16859633, rs9288953, rs2705535, rs1844089, rs2705565, and rs2633580, and CLL risk was evaluated in 321 patients and 470 HC in the Polish population. Among those SNPs studied rs76844316 and rs16859633 were not detected in either group. The global distribution of rs2705511, rs1982809, and rs9288953 differed significantly between CLL and HC. The frequency of rs1982809 GA and GG genotypes and the rs2705511 CC genotype was higher in CLL patients compared to HC. In addition, carriers of the rs2705511 C allele and the rs1982809 G allele were more prone to develop CLL by 1.6 and 1.5 times, respectively. Also, individuals with the rs9288953 TT genotype had twice higher CLL risk compared to carriers of the rs9288953 CC genotype. The global distribution of haplotypes differed significantly between CLL patients and HC, where the CGATTCGCC (rs2705511, rs1982809, rs9288952, rs9288953, rs2705535, rs1844089, rs2705565, rs2633580) haplotype significantly increased risk of CLL by 1.6 times. Additionally, we studied the correlation between the presence of *BTLA* SNPs with BTLA expression level in CLL patients. Carriers of the rs1982809 G allele (GG + GA) had lower median BTLA mRNA expression levels in T-cells compared to the rs1982809 AA genotype. The presence of rs1982809 had no influence on BTLA expression levels in B-cells of CLL patients [118].

### Lung cancer

To date, the association of *BTLA* polymorphisms with lung cancer susceptibility has been studied in Caucasian, Tunisian and Chinese populations. In the Tunisian population, Khadhraoui et al. investigated the association of three *BTLA* SNPs: rs1982809, rs9288952, and rs9288953 in 169 lung cancer patients and 300 HC. Khadhraoui’s group noted that the frequencies of the rs1982809 AG genotype and the G allele were significantly higher among patients compared to HC and increased lung cancer risk by 1.6 and 1.4 times, respectively. Haplotype analysis showed differences in haplotype distribution between patients and HC, however after applying Bonferroni correlation this association lost significance. With regard to age, gender, smoking status, and metastases status statistical analysis failed to reveal any association of *BTLA* SNPs with lung cancer risk. The rs1982809 AG genotype was associated with bigger tumor size (OR = 1.8), lymphatic invasion (OR = 3.7), as well as with the development of the adenocarcinoma subtype (OR = 2.8) in patients. Survival analysis did not show any correlation between the *BTLA* SNPs studied and patient survival [121].

In one of our studies by Andrzejczak et al. we investigated the correlation between seven *BTLA* SNPs: rs2705511, rs1982809, rs9288952, rs9288953, rs1844089, rs11921669, and rs2633582, and susceptibility to NSCLC in 383 cases and 474 HC in the Polish population. It was found that the rs1982809 SNP was associated with NSCLC risk, where carriers of the rs1982809 G allele were more frequent in NSCLC patients compared to HC [122]. Our results were consistent with Khadhraoui’s study [121]. After analysis with clinical data, the rs1982809 G allele carriers were overrepresented in never-smokers, but not in smokers, compared to HC. In addition, haplotype global distribution differed between never-smokers and smokers, where CGACG, CGATG, and AGGCA (rs2705511, rs1982809, rs9288952, rs9288953, rs1844089) haplotypes were more frequent in never-smokers. After stratification by gender, females carrying the rs1982809 G allele or the rs9288953 T allele had a higher risk of NSCLC by 1.6 and 2.2 times, respectively When disease stage was taken in to consideration, rs1982809 G and rs2705511 C alleles were associated with the more advanced stages of NSCLC (stage II and III), but not with stage IV [122]. Moreover, similarly to Khadhraoui’s study [121], we noticed a higher risk of adenocarcinoma in patients bearing the rs1982809 AG genotype. The Kaplan-Meier analysis revealed that rs1982809 and rs2705511 significantly modified patient OS [122].

Wang et al. aimed to identify the association of *BTLA* SNPs with the risk of NSCLC development in the Chinese population. In this study, four *BTLA* SNPs: rs1982809, rs16859629, rs2171513, and rs3112270 were genotyped in 1003 NSCLC patients and in 901 HC. In contrast to the two abovementioned studies, Wang’s group documented in the Chinese population that in comparison to carriers of the rs1982809 GG genotype the presence of the rs1982809 A allele (GA and AA + GA) increased the risk of NSCLC in those patients. Furthermore, after subgroup analysis, the authors noticed that the G to A change of the rs1982809 genotype reduced the overall risk of NSCLC, especially in the non-SCC, BMI ≥ 24 kg/m^2^, ≥ 59 year, and never-drinking patients. Additionally, the authors noticed that rs3112270 GG + AG genotypes decreased susceptibility to non-squamous cell carcinoma (non-SCC) compared to the AA genotype. Whereas, rs16859629 CC and rs2171513 AA genotypes may promote the development of SCC [123].

Comparing the results from the three abovementioned studies, a discrepancy between results regarding rs1982809 can be noticed. Moreover, in other cancers, we also observed conflicting results for this polymorphism. In the Chinese population, possession of the A allele or the AA genotype is potentially associated with an increased risk of disease, while in other populations carriers of the G allele or the GG genotype seem to be more prone to cancer development. This discrepancy may result from big differences in the rs1982809 frequency in the Asian population (excluding South Asian) (A = 25–35%; G = 75 − 65%), compared to other populations including Caucasians, where the distribution of rs1982809 alleles is similar (A = 60–77%; G = 40 − 23%) [114].

Of equal importance, subgroup analysis of much more homogenous cohorts can reveal associations not noticed in heterogenous groups. This can be clearly seen for example when NSCLC patients were divided into subgroups based on histological subtype [122, 123]. Similarly, smoking status may be the factor masking the influence of low penetrating risk factors like *BTLA* SNPs, which was found for NSCLC [122] and EGJA [124].

### Gastrointestinal cancers

Association between *BTLA* polymorphisms and the risk of CRC in the Chinese population has been investigated by Ge et al. They genotyped three *BTLA* SNPs: rs1844089, rs2705535, and rs9288935 in 601 cases and 627 HC. The presence of the rs2705535 TT genotype was associated with an increased risk of rectal cancer by 1.8 times, while the rs9288953 TT genotype decreased this risk by 1.4 times when compared to wild alleles carriers. Haplotype analysis did not reveal any significant differences in the haplotype distribution between patients and HC. In another study, Tang et al. evaluated the influence of four *BTLA* SNPs: rs16859629, rs1982809, rs2171513, and rs3112270 on EGJA risk in 1234 patients and 1540 HC in the Chinese population. None of SNPs studied appeared to be associated with the risk of EGJA. Nevertheless, subgroup analysis indicated that the presence of the rs1982809 AA genotype was correlated with 2 times higher EGJA risk in heavy smokers. Moreover, haplotype analysis showed that the TAAG (rs16859629, rs1982809, rs2171513, rs3112270) haplotype correlated with a three times higher risk of EGJA development [124]. Similarly, Cao et al. who genotyped four *BTLA* SNPs: rs16859629, rs1982809, rs2171513, and rs3112270 in 721 cases and 1208 HC in the Chinese population, did not find any significant relationship between *BTLA* polymorphisms and esophageal squamous cell carcinoma (ESCC) in their OS. However, after adjustment by age, sex, BMI, smoking status, and alcohol consumption it was observed that in individuals who overused alcohol the presence of the rs2171513 A allele was associated with a 1.6 times lower risk of ESCC. Moreover, in overweight subjects (BMI ≥ 24) the presence of the rs3112270 GG genotype was associated with 1.9 times increased risk of ESCC compared to the AA carriers [125].

### Renal cancer

There has been only one study investigating the role of *BTLA* SNPs in RCC. In our study, we aimed to investigate the association between *BTLA* SNPs and RCC susceptibility in the Polish population, seven *BTLA* SNPs; rs1844089, rs2705535, rs9288953, rs9288952, rs16859633, rs1982809, and rs2705511 were genotyped in 282 RCC patients and 480 HC. One of the studied SNPs, rs16859633, was found to be absent in the Polish population but present in the Asian population. We found that the presence of the rs1982809 G allele increased RCC risk by 1.4 times. Furthermore, carriers of rs1982809 G and rs2705511 C alleles had a 40% increased RCC risk compared to individuals possessing the AA genotype for both SNPs. Additionally, the presence of the CGATCG (rs2705511,rs1982809 rs9288952, rs9288953, rs2705535, rs1844089) haplotype increased RCC risk by 1.5 times. In the subgroup analysis, patients with high-grade ccRCC had a higher frequency of the rs1982809 GG genotype compared to low-grade ccRCC patients and to HC [126].

**Table 4:** Summary of the reported relationships between single nucleotide polymorphisms located in the *BTLA* gene and cancer risk

Group [Ref]	Cancer type	Population	Studied SNPs	Associations found
Fu et al. [119]	BC	Chinese	rs9288952 rs2931761 rs2633562 rs2705535 rs1844089	Genotype distributions of rs1844089, rs2705535 and rs9288952 differed between BC patients and HC; rs1844089 CT genotype increased BC risk by 1.3 times, CC genotype decreased BC risk 1.3 times; rs2705535 CT genotype increased BC risk by 1.5 times, CC genotype decreased BC risk 1.5 times; rs9288952 GG genotype lowered BC risk 1.7 times; Haplotype GTTTT (rs9288952, rs2931761, rs2633562, rs2705535, rs1844089) was associated with 3 times increased BC risk and haplotype GTCTC was more frequent in patients with lymph node invasion;
Zhao et al. [120]	BC	Chinese	rs1982809	rs1982809 AA genotype was associated with 1.95 times higher BC risk after adjustment of BMI, age, and menopausal status compared to GG genotype; rs1982809 was associated with the ER status, Ki-67 status, TNM stage and tumor size of BC;
Karabon et al. [118]	CLL	Caucasian	rs2705511 rs1982809 rs9288952 rs76844316 rs16859633 rs9288953 rs2705535 rs1844089 rs2705565 rs2633580	rs1982809 G allele (AG + GG genotypes) was associated with 1.5 times higher CLL risk; rs2705511 C allele (AC + CC genotypes) was associated with 1.5 times higher CLL risk; rs9288953 TT genotype was associated with 2 times higher CLL risk and the CT genotype with 25% higher risk of CLL; Haplotypes distribution differed significantly between CLL patients and controls; Haplotype CGATCGCC (rs2705511, rs1982809, rs9288952, rs9288953, rs2705535, rs1844089, rs2705565, rs2633580) increased CLL risk and haplotype AAACCGCC decreased the risk of CLL; rs1982809 G allele (AG + GG genotype) was associated with lower BTLA mRNA expression level in T-cells compared to AA genotype in CLL patients
Khadhraoui et al. [121]	lung cancer	Tunisian	rs1982809 rs9288952 rs9288953	rs1982809 G allele (AG + GG genotypes) was associated with 1.5 times higher NSCLC risk; rs1982809 was associated with T4 tumor size, lymph node invasion and adenocarcinoma subtype; Haplotypes were differently distributed in patients than in controls;
Andrzejczak et al. [122]	NSCLC	Caucasian	rs2705511 rs1982809 rs9288952 rs9288953 rs1844089 rs11921669 rs2633582	rs1982809 G allele (AG + GG genotypes) was associated with 1.3 times higher NSCLC risk; rs9288953 genotype distribution differed between female patients and female controls and rs9288953 T allele (CT + TT genotypes) was associated with 2.2 times higher NSCLC risk in females; rs1982809 genotypes distribution differed between non-smokers and controls, and rs1982809 A allele (AA + AG genotypes) decreases susceptibility to NSCLC by 3 times; rs1982809 G and rs2705511 C alleles correlated with the more advanced stages of NSCLC; Haplotype distribution differed significantly between never-smokers and controls; rs1982809 and rs270551 significantly modified patient survival (OS);
Wang et al. [123]	NSCLC	Chinese	rs1982809 rs16859629 rs2171513 rs3112270	rs3112270 G allele (GG + AG genotype) decreased NSCLC risk; rs1982809 GA and AA + GA genotypes were associated with NSCLC risk; rs2171513 AA and rs16859629 CC genotypes increased squamous cell carcinoma (SCC) risk; Haplotype TAGG (rs16859629, rs1982809, rs2171513, rs3112270) reduced NSCLC risk compared to TGGA;
Ge et al. [116]	CRC RCa	Chinese	rs3087243 rs231775 rs231777 rs1844089 rs2705535 rs10204525 rs2227982 rs9288953 rs7421861 rs6710479	rs2705535 TT genotype was associated with 2 times higher CRC risk compared to the CC genotype; rs9288953 TT genotype decreased RCa risk by 1.4 times;
Tang et al. [124]	EGJA	Chinese	rs16859629 rs1982809 rs2171513 rs3112270	No association was found between BTLA SNPs and EGJA risk in the overall analysis; Haplotype TAAG (rs16859629, rs1982809, rs2171513, rs3112270) increased EGJA risk by 3 times; rs1982809 AA genotype was associated with 2 times higher EGJA risk in heavy-smokers;
Cao et al. [125]	ESCC	Chinese	rs2171513 rs3112270 rs1982809 rs16859629	No association was found between BTLA SNPs and ESCC risk in the overall analysis; rs3112270 CT genotype reduced ESCC risk in male patients by 1.2 times; rs3112270 CC genotype increased ESCC risk in BMI ≥ 24 kg/m2 patients’ group by 2 times; rs2171513 AG genotype, when compared to AA genotype, reduced ESCC risk by 1.6 times in the ever-drinking patients;
Partyka et al. [126]	RCC	Caucasian	rs1844089 rs2705535 rs9288953 rs9288952 rs16859633	rs1982809 G allele (AG + GG genotypes) was associated with 1.4 times higher RCC risk; Overrepresentation of rs2705511 C allele carriers (dominant model) in RCC patients; Haplotype CGATCG (rs2705511, rs1982809, rs9288952, rs9288953, rs2705535, rs1844089) increased RCC risk by 46%; rs1982809 GG genotype was associated with 2.75 times higher risk of High Grade ccRCC; Genotype distribution of rs2705535 in High Grade ccRCC significantly differed compared to controls; rs9288953 TT genotype tended to be more frequent in High Grade ccRCC than in controls

HC - healthy controls, BC - breast cancer, CLL - chronic lymphocytic leukemia, NSCLC - non-small-cell lung cancer, CRC - colorectal cancer, RCa - rectal cancer, EGJA - esophagogastric junction adenocarcinoma, ESCC - esophageal squamous cell carcinoma, RCC - renal cell carcinoma

## BTLA‑targeted therapies in clinical trials

Understanding BTLA’s role in cancer has led to the exploration of therapeutic strategies targeting this pathway. Blockade of the BTLA-HVEM axis using mAbs has shown promising results in preclinical models, enhancing anti-tumor immune responses leading to tumor regression and improved survival. Additionally, combining BTLA blockade with other IC inhibitors has shown synergistic effects, further highlighting the therapeutic potential of targeting BTLA [5, 69, 97].

Icatolimab ((TAB004/JS004)) the world´s first-in-class anti-BTLA humanized IgG4 mAb was approved in 2019 by the FDA for clinical trials. This anti-BTLA mAb contains a hinge mutation (S228P) that binds BTLA and blocks its interaction with HVEM. It is currently being tested in phase I and II clinical trials in the treatment of advanced and metastatic solid tumors and lymphomas, alone or in combination with anti-PD-1 mAb (toripalimab, JS001), (summarized in Table 5). In the first-in-human dose-escalation phase Ia study (NCT04137900) 25 patients with advanced solid tumors with a median of 4 prior lines of therapy were enrolled, including patients who progressed upon prior anti-PD-1/L1 therapy. Preliminary data showed good tolerance and clinical efficacy of icatolimab monotherapy. Among 19 evaluable patients, one partial response (PR) (melanoma) and six stable diseases (SD) were documented (with a median follow-up of 32 weeks). Analysis of biomarkers revealed that a favorable response was linked to the concurrent expression of HVEM and CD8 [127].

In another phase I/II clinical trial (NCT05000684), safety and anti-tumor activity of tifcemalimab (anti-BTLA) in combination with toripalimab treatment were evaluated. For this purpose, 43 patients with extensive-stage small-cell lung cancer (ES-SCLC) refractory to prior therapies (among others to anti-PD-1/L1 treatment) were enrolled. Study objectives included safety, anti-tumor activity and correlative biomarkers. Preliminary results suggest the safety of the combined BTLA and PD-1 blockade is promising. The overall response rate was 26.3% and the disease control rate was 57.9% in 38 efficacy evaluable patients. Tumor expression of HVEM and PD-L1 was evaluated as a biomarker of clinical response. After a median follow-up of 12.1 weeks, 70.0% of the responses were ongoing, however, the median duration of the response was not reached [128]. Therefore, further evaluation of this combinational treatment in ES-SCLC is essential.

The usefulness of BTLA blockade is also assessed in the treatment of hematological malignancies. In an ongoing phase I study (NCT04477772) 48 patients with relapsed/refractor lymphoma, including 28 HL and NHL, were enrolled. All patients underwent a median of 4 prior therapy lines, including patients who progressed upon prior anti-PD-1/L1 therapy. Study objectives included safety, pharmacokinetics, and efficacy. Among the patients treated, 25 received tifcemalimab as monotherapy and 23 combined anti-BTLA/PD-1 therapy. No dose-limiting toxicity was observed in either monotherapy or combination dose escalation. Monotherapy results showed one PR (FL) and seven SD among 22 evaluable patients, with a median follow-up of 31.3 weeks. Further, among 12 HL evaluable patients treated with combined anti-BTLA/PD-1 therapy one complete response (CR), four PR, and five SD were observed. In general, tifcemalimab alone or in combination with toripalimab showed good tolerance and promising clinical efficacy in relapsed/refractor lymphomas. Additionally, preliminary data suggests a discernible correlation trend between elevated HVEM expression and clinical responsiveness [129]. Although the available data show promising results, further studies on the safety and effectiveness of BTLA blockade alone or in combination with other IC inhibitors is needed.

**Table 5:** Summary of clinical trials targeting BTLA in cancer

Clinical Trial	Year	Cancer	Phase	Therapy	Status
NCT04137900	2019	Lymphoma, melanoma, NSCLC, RCC, UC or other tumors	Phase I	Monotherapy or combined with Toripalimab	Recruiting
NCT04278859	2020	Advanced solid tumors	Phase I	Monotherapy	Unknown
NCT04477772	2020	Recurrent/refractory malignant lymphoma	Phase I	Monotherapy or combined with Toripalimab	Active, not recruiting
NCT04773951	2021	Melanoma, RCC, UC	Phase I	Monotherapy or combined with Toripalimab	Recruiting
NCT04929080	2021	HNSCC, NPC	Phase I/II	Monotherapy or combined with Toripalimab	Not recruiting, completed
NCT05000684	2021	Advanced lung cancer	Phase I/II	Monotherapy or combined with Toripalimab	Recruiting
NCT05427396	2022	LC, ESCC, GC, cervical cancer, CRC	Phase I	Combined with Toripalimab	Recruiting
NCT05664971	2022	Advanced lung cancer	Phase Ib/II	Combine with Toripalimab and with chemotherapy	Recruiting
NCT05789069	2023	RCC, melanoma, NSCLC, GC, CRC	Phase Ia/b	Monotherapy or in combination with tislelizumab	Recruiting
NCT05891080	2023	Stage III NSCLC	Phase II	Combine with Toripalimab and with chemotherapy	Not yet recruiting

NSCLC - non-small-cell lung cancer, RCC - renal cell carcinoma, UC - urothelial carcinoma, HNSCC - head and neck squamous cell carcinoma, NPC - nasopharyngeal carcinoma, LC - liver cancer, ESCC - esophageal squamous cell carcinoma, GC - gastric cancer, CRC - colorectal cancer

## Conclusions

BTLA is a crucial immune checkpoint receptor that regulates immune responses. Recent investigations emphasize its significance in the context of cancer, making it a promising therapeutic target and a valuable prognostic marker. Notably, in specific malignancies such as hepatocellular carcinoma, lung cancer, and melanoma, BTLA expression is upregulated on TILs, indicating an immune escape mechanism employed by cancer cells. The heightened BTLA expression on TILs leads to T-cell exhaustion, reducing their capacity to eliminate cancer cells effectively. Furthermore, in certain cancer types like gastric and breast cancer, elevated BTLA levels correlate with poor prognosis, suggesting its role as a prognostic marker. Additionally, BTLA expression levels hold the potential to predict immunotherapy response, although elevated expression in some tumors can cause resistance to such treatments. Promisingly, preclinical investigations focused on blocking the BTLA-HVEM axis have demonstrated encouraging results in enhancing anti-tumor immune responses, leading to tumor regression. These findings are further supported by Phase I clinical studies positive results. Therefore, BTLA emerges as a promising therapeutic target and prognostic marker in cancer, with its modulation offering potential avenues for enhancing anti-tumor immune responses and improving clinical outcomes.

## Data Availability

Not applicable.

## Abbreviations

ICs: Immune checkpoints
BTLA: B and T lymphocyte attenuator
sBTLA: Soluble BTLA
HVEM: Herpes virus entry mediator
TNFR: Tumor necrosis factor receptor
CDR: Complementarity-determining region
Grb2: Growth-factor receptor-bound protein 2
ITIM: Immunoreceptor tyrosine inhibitory motif
ITSM: Immunoreceptor tyrosine-based switch motif
Y257: Tyrosine 257
Y282: Tyrosine 282
APC: Antigen presenting cells
DC: Dendritic cells
PBMCs: Peripheral blood mononuclear cells
Tfh cells: T follicular helper cells
Abs: Antibodies
PB: Peripheral blood
BLNK: B-cell linker protein
PLCγ2: Phospholipase Cγ2
TB: Tuberculosis
TME: Tumor microenvironment
TILs: Tumor-infiltrating lymphocytes
SRSF2: serine/arginine-rich splicing factor 2
STAT3: activator of transcription 3
IHC: Immunohistochemistry
NHL: Non-Hodgkin lymphomas
B-SLL: B-cell small lymphocytic lymphoma
MCL: Mantle cell lymphoma
FL: Follicular lymphoma
DLBCL: Diffuse large B-cell lymphoma
MZL: Marginal zone lymphoma
BL: Burkitt lymphoma
HL: Hodgkin lymphomas
CLL: Chronic lymphocytic leukemia
PTCL: Peripheral T-cell lymphoma
HC: Healthy controls
OS: Overall survival
TTT: Time to treatment
NSCLC: Non-small cell lung cancer
RFS: Recurrence-free survival
TCGA: Cancer Genome Atlas
EMT: Epithelial-mesenchymal transition
LUAD: Lung adenocarcinoma
TMA: Tumor antigens
ACT: Adoptive cell therapy
MAFs: Melanoma-associated fibroblasts
SKCM: Skin cutaneous melanoma
DSS: Disease-specific survival
DFI: Disease-free interval
GC: Gastric cancer
CRC: Colorectal cancer
PFI: Progression-free interval
TI: Tumor-infiltrating
IF: Immunofluorescence
XGC: Xanthogranulomatous cholecystitis
CC: Chronic cholecystitis
HCC: Hepatocellular carcinoma
OSCC: Oral and squamous cell carcinoma
TMB: Tumor mutation burden
NOM: Healthy oral mucosa
DTC: Differentiated thyroid carcinoma
TC: Thyroid carcinoma
EOC: Epithelial ovarian cancer
BC: Breast cancer
PyMT: Polyoma middle T oncogene
GBM: Glioblastoma
GME: Glioma microenvironment
HGG: High-grade glioma
FC: Flow cytometry
RS: RNA-seq data
NS: NanoString
RCC: Renal cell carcinoma
ccRCC: Clear cell renal cell carcinoma
PDAC: Pancreatic adenocarcinoma
PCa: Prostate cancer
PFS: Progression-free survival
HNCa: Head and neck cancer
ER: Estrogen receptor
PR: Progesterone receptor
non-SCC: non-squamous cell carcinoma
ESCC: Esophageal squamous cell carcinoma
PR: Partial response
SD: Stable disease
ES-SCLC: Extensive-stage small-cell lung cancer
CR: Complete response
UC: Urothelial carcinoma
HNSCC: Head and neck squamous cell carcinoma
NPC: Nasopharyngeal carcinoma
LC: Liver cancer

## Footnotes

1 In the original manuscript, there were no spaces between the protein name and the sign + or -. In this case, + stands for positive, and - for negative. Currently, once there is a space and sometimes there is no space, for example, BTLA + CD8+/CD4 + or BTLA-CD3 +. Please standardize this in the whole manuscript (there are many like this).
